# Supplementary material for: Identification of age-specific gene regulators of La Crosse virus neuroinvasion and pathogenesis
Source: Nat Commun. 2023 May 18;14:2836. doi: 10.1038/s41467-023-37833-x (PMC10195820; doi:10.1038/s41467-023-37833-x)
Supplement: Supplementary file 1 — Supplementary Information [file 41467_2023_37833_MOESM1_ESM.pdf]

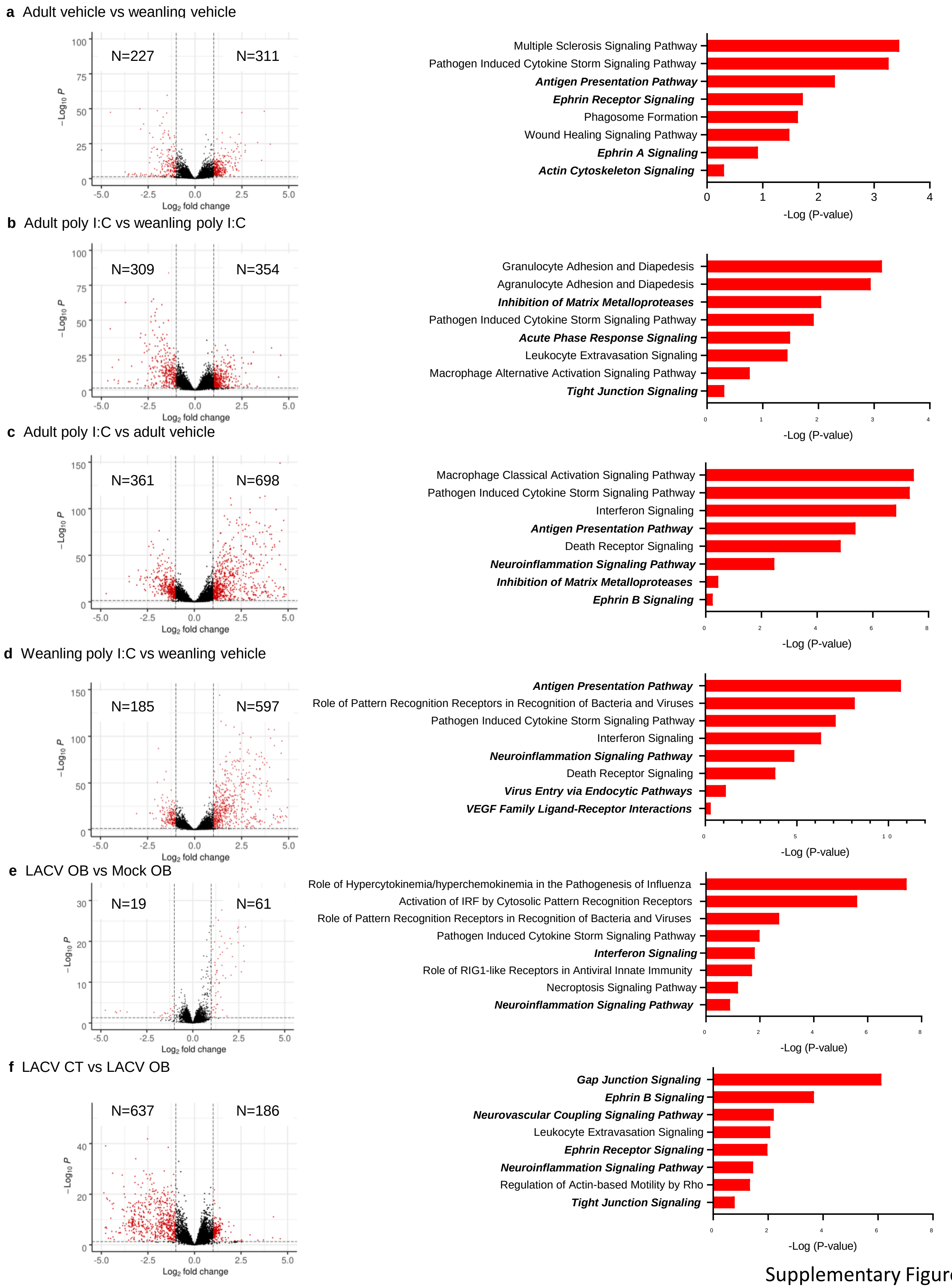

Supplementary Figure 1

Supplementary Figure 1 continued

Supplementary Figure 1 (related to Figure 1). Differentially expressed genes and pathway enrichment analyses from RNA-seq.

(A-F) Left panels: Volcano plots show the differentially expressed genes in BCECs comparing (A) adult vehicle vs weanling vehicle, (B) adult poly I:C vs weanling poly I:C, (C) adult poly I:C vs adult vehicle, (D) weanling poly I:C vs weanling vehicle, (E) LACV olfactory bulb (OB) vs mock OB and (F) LACV cortex (CT) vs LACV OB. Genes with a basemean ≥100 with log fold change ≥ 2 and P<0.05 are shown in red and genes not meeting these criteria are shown as black. (A-F) Right panels: Utilizing IPA software, the pathways with the most significant difference between the indicated pairwise comparisons are shown. Analyses performed on RNA-seq samples from adult and weanling poly I:C treated BCECs are shown in A-D, whereas the analyses comparing BCECs from weanling, LACV infected OB v. CT BCECs are in E and F. The most relevant pathways from the IPA analysis are represented in bold and italicized font in the right side graphs. Differential gene expression analysis was performed using the DESeq2 statistical package. The default DESeq function with betaPrior=FALSE was applied, wherein a negative binomial generalized model with Wald test for significance was performed. The two-sided p-values were corrected for multiple testing using the Benjamini Hochberg method.

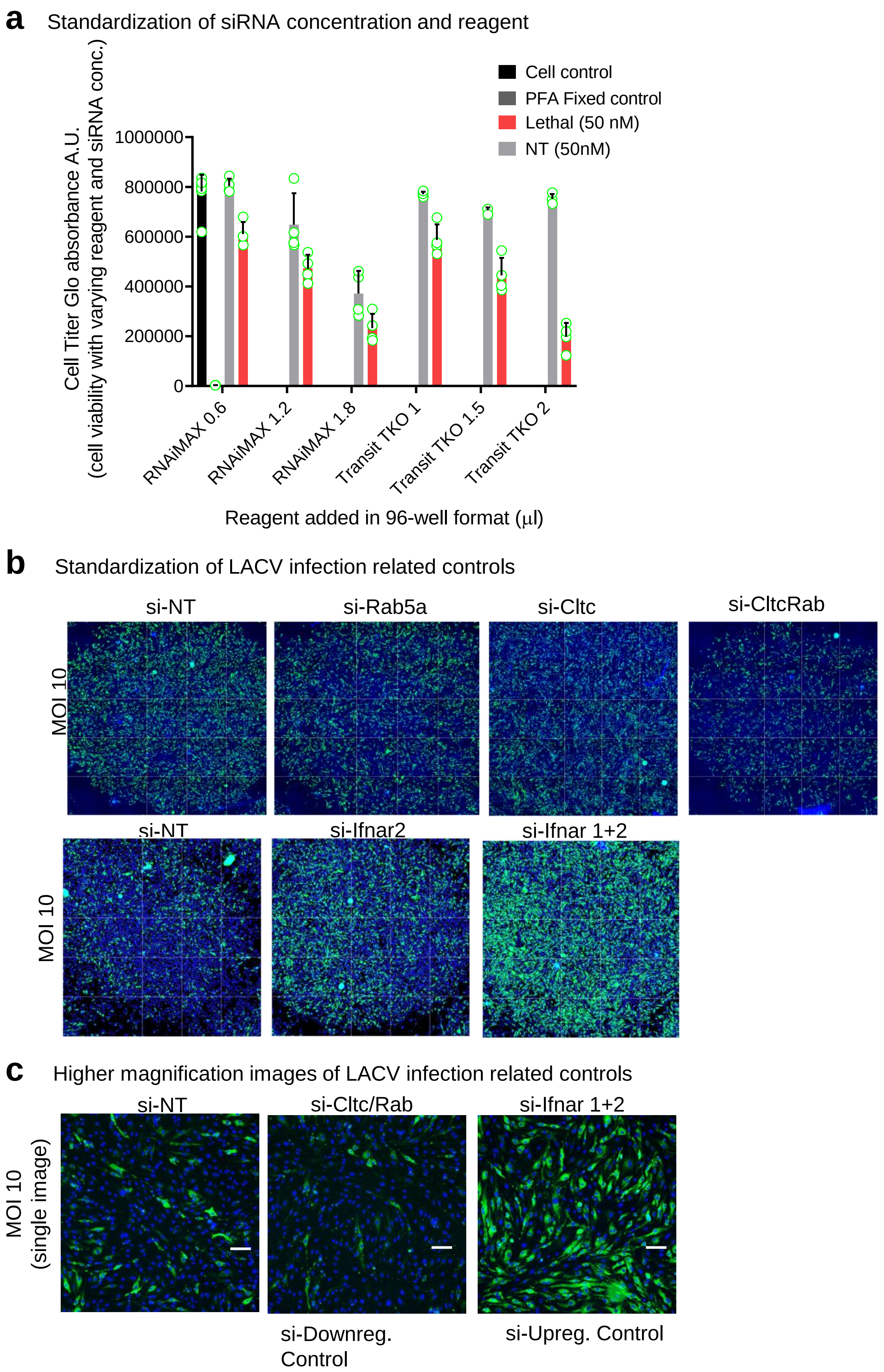

Supplementary Figure 2

Supplementary Figure 2 continued

Supplementary Figure 2 (related to Figure 2). Standardization of siRNA transfection in bEnd.3 cells.

(A) bEnd.3 cells' viability is shown for different siRNA transfection methods using RNAiMAX or Transit TKO (amount of transfection reagent/well is indicated in X-axis labels) in a 96 multi-well plate format at 72 hpi. For each condition, si-NT (light grey) was compared with si-Lethal (red) (N=4-8, mean +/- SD is shown). (B) The infection of bEnd.3 cells (10MOI LACV) is shown for different siRNA treatment groups from an entire well of cells to demonstrate establishment of appropriate controls. Viral downregulation control (si-CltcRab (siRNA against Clathrin heavy chain (si-Cltc) and against Rab5a (si-Rab) were deployed together)) and viral upregulation control (si-Ifnar1+2 (siRNA against Ifnar1 and against Ifnar2 were deployed together). Non-targeting (si-NT) was used as a baseline (LACV: green and Hoechst (nuclei): blue). (C) Higher magnification image of bEND.3 cells transfected with different control classes. Images are representative of 3 experiments. Scale bar= 100 µm for bottom panel. Merged images from entire well plate is shown for rest of the panels.

a Representative images of 6 hpi siRNA screen (changes in LACV stain post-siRNA treatment)

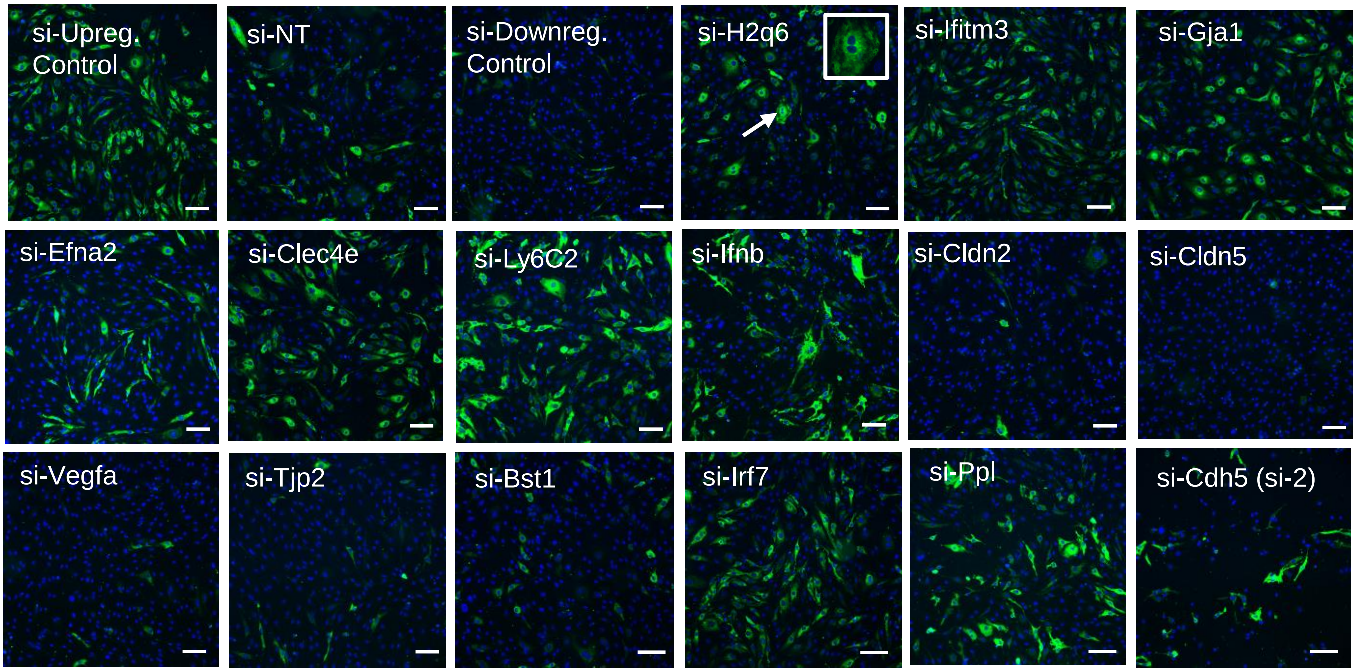

b Representative images of 24 hpi siRNA screen (changes in LACV stain post-siRNA treatment)

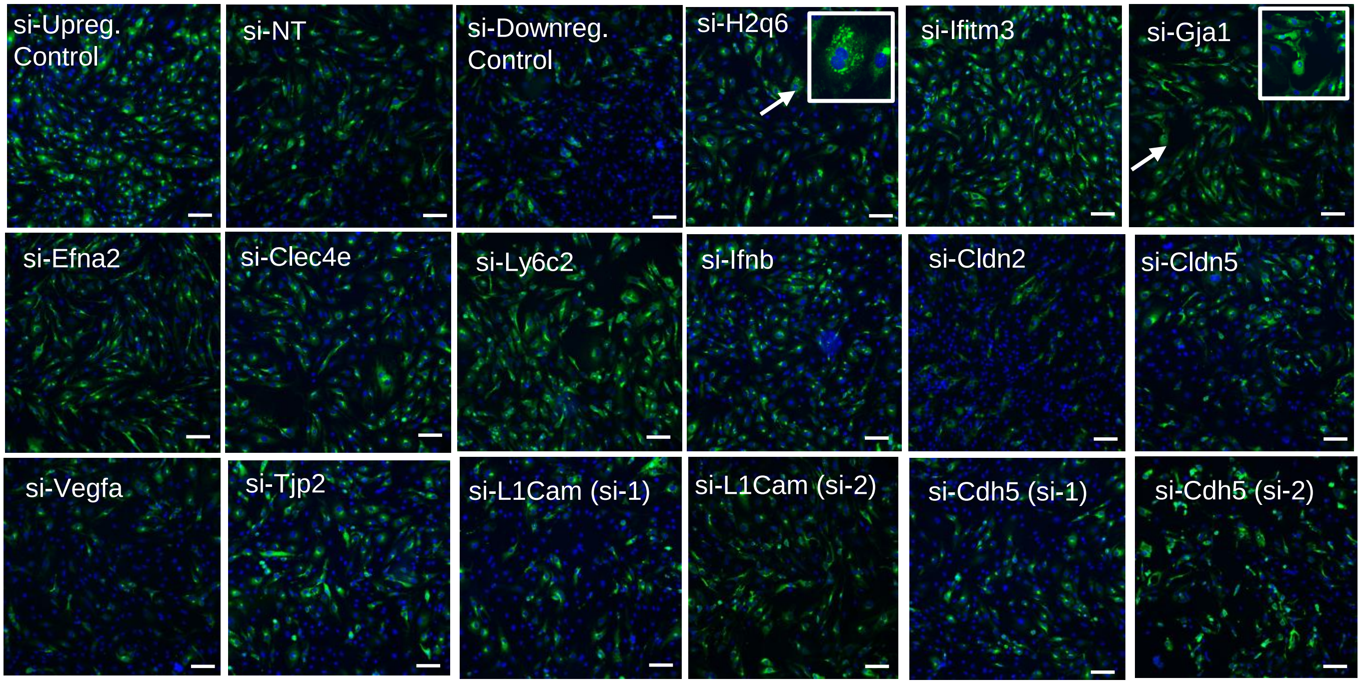

Supplementary Figure 3

Supplementary Figure 3 continued

Supplementary Figure 3 (related to Figure 2 and 3). Representative images of select gene hits from the targeted siRNA targeted screen.

(A-B) bEND.3 cells were transfected with 50 nM of the indicated gene specific siRNA for 72 hr, infected with 10 MOI LACV, and imaged for viral infection intensity at (A) 6 hpi or (B) 24 hpi (LACV (green) and Hoechst (blue). Representative images from replicate screens are shown for several target genes compared to controls (25 images acquired per condition). The arrows represent cytopathic effects after specific gene knockdown where the syncytia-like aggregate formation is represented in the insets. Some genes showed opposing effects between different siRNA (shown as si-1 or si-2). Scale bar=100 µm.

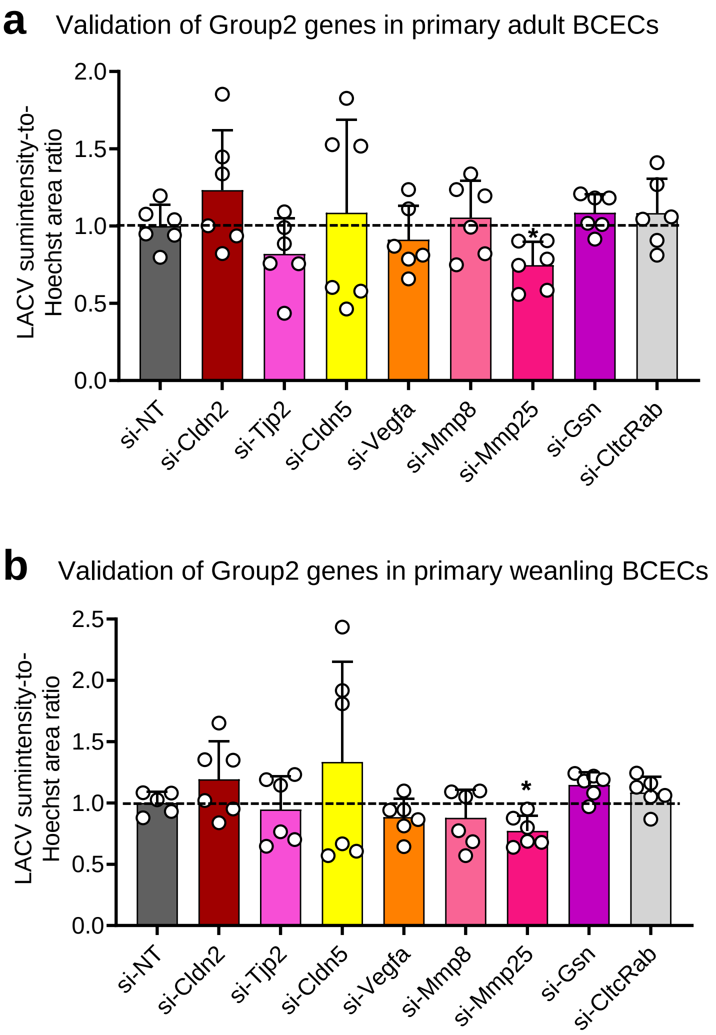

Supplementary Figure 4 (related to Figure 3). Group2 genes show marginal effect on LACV infection in primary BCECs.

Adult (A) and weanling (B) primary BCECs were transfected with 50 nM siRNA for 72 hr against the indicated Group2 hit genes (mainly susceptibility factor candidates) and LACV infection (LACV sum-intensity-to-Hoechst area ratio) was assessed (N=5-6 for both A and B, where individual datapoints are shown, P=0.2075, 0.1885, 0.7488, 0.4797, 0.6326, 0.0290, 0.1988 and 0.5603 in Panel A and P=0.1650, 0.9000, 0.7383, 0.3229, 0.5459, 0.0222, 0.1282 and 0.3247 in Panel B for si-Cldn2, si-Tjp2, si-Cldn5, si-Vegfa, si-Mmp8, si-Mmp25, si-Gsn and si-Downreg. Control compared to si-NT). Multiple paired, two-tailed t-tests between si-NT and the targeted genes were performed (*P<0.05) and mean ± SD are shown.

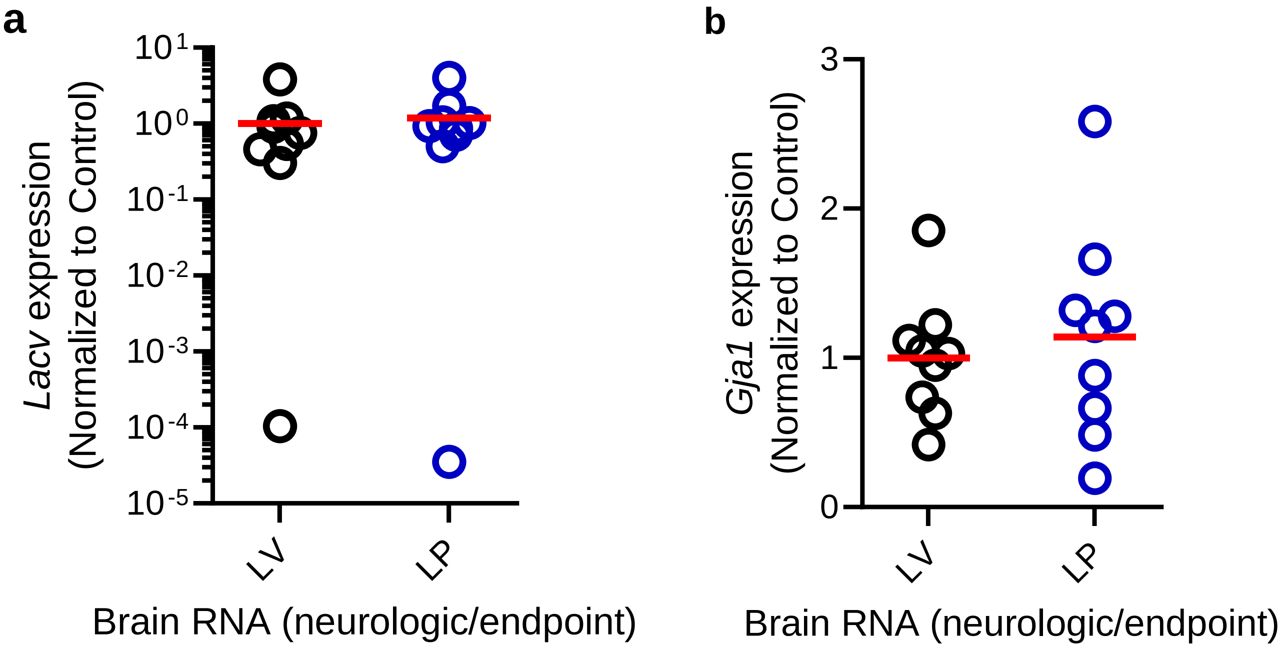

Supplementary Figure 5 (related to Figure 6). No significant differences observed in LV and LP mice at the neurological endpoint.

(A-B) Weanling mice, infected with 2000 PFU of LACV, were treated with vehicle (LV) or 500mg/kg-day 4-PBA (LP) until 5 dpi then assessed for neurologic endpoint. RNA was isolated from mouse brains at the endpoint defined by clinical disease and expression levels of (A) LACV RNA (N=9 animals, P=0.7160, unpaired, two-tailed t-test) and (B) Gja1 mRNA (N=9 animals, P=0.6109, unpaired, two-tailed t-test) were assessed by qPCR.

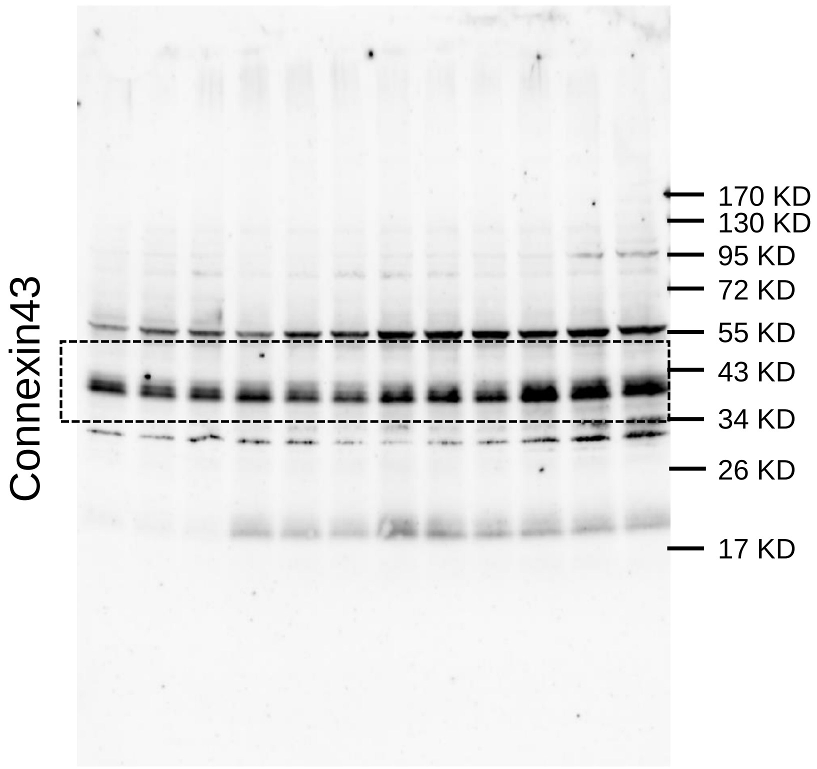

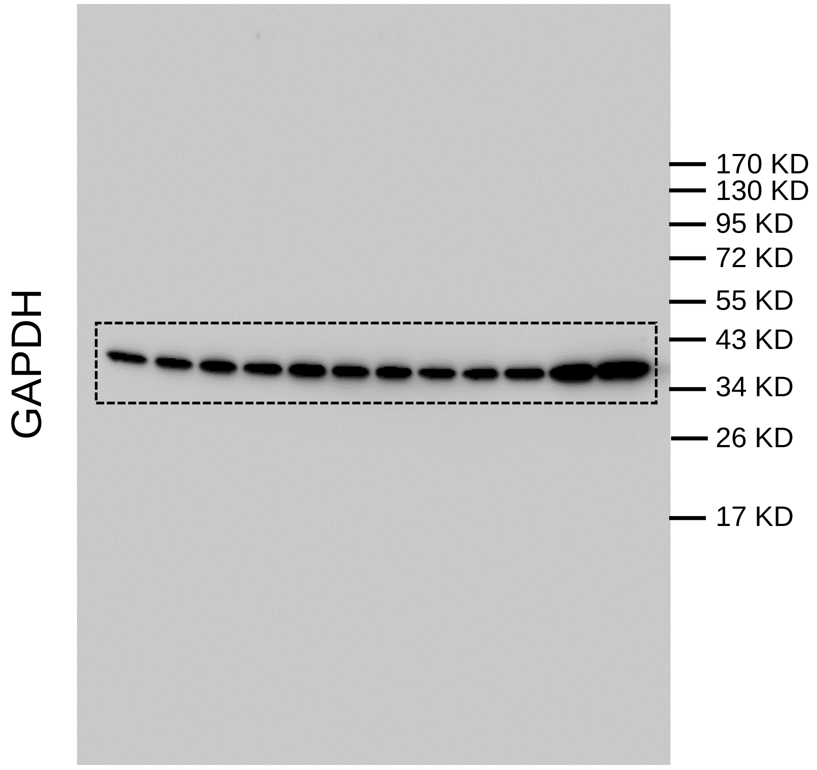

Supplementary Figure 6 (related to Figure 6). Unprocessed blot data.

The unprocessed western blot data are provided, along with the protein marker bands.

Supplementary Table 1

Gene abbreviation	Gene name	Selection criteria	Groups	Log 2 fold change	P (adj) value
Bst1	Bone marrow stromal cell antiger	High AV versus WV expression	Group 1: Poly I:C-Vehicle RNA-Seq	-3.72 (AV vs WV)	2.40000E-04
Efna2	Ephrin A2	High AV versus WV expression		2.25 (AV vs WV)	9.32911E-23
H2q6	Histocompatibility 2, Q region loc	High AV versus WV expression		2.66 (AV vs WV)	2.00513E-17
L1cam	L1 cell adhesion molecule	High AV versus WV expression		2.34 (AV vs WV)	1.94542E-17
Mmp8	Matrix metallopeptidase 8	High AV versus WV expression		-4.68 (AV vs WV)	1.69503E-04
Mmp15	Matrix metallopeptidase 15	High AV versus WV expression		-1.99 (AV vs WV)	4.28395E-15
Mmp25	Matrix metallopeptidase 25	High AV versus WV expression		-1.72 (AV vs WV)	7.26597E-16
Il10	Interleukin 10	High AV versus WV expression (low base-mean)		-2.39 (AV vs WV)	2.33081E-02
Clec4e	C-type lectin domain family 4, me	High API versus AV expression		4.80 (API vs AV)	1.42984E-05
Cxcl10	Chemokine (C-X-C motif) ligand	High API versus AV expression		4.76 (API vs AV)	4.20082E-85
Ifitm3	Interferon induced transmembrar	High API versus AV expression		2.68 (API vs AV)	2.07954E-10
Irf7	Interferon regulatory factor 7	High API versus AV expression		4.75 (API vs AV)	2.69890E-14
Ifna4	Interferon alpha 4	High API versus AV expression (low base-mean)		5.85 (API vs AV)	8.41505E-03
Cdh1	Cadherin 1	High API versus WPI expression		3.30 (API vs WPI)	3.64819E-08
Ccl11	Chemokine (C-C motif) ligand 11	High API versus WPI expression		2.99 (API vs WPI)	3.39982E-17
Cdhr1	Cadherin-related family member	High API versus WPI expression (low base-mean)		5.43 (API vs WPI)	3.59312E-04
Ly6c2	Lymphocyte antigen 6 complex, l	High API versus WPI expression		2.90 (API vs WPI)	4.19378E-04
Aqp1	Aquaporin 1	High LCT versus LOB expression	Group 2: OB-CT RNA-Seq	2.32 (LCT vs LOB)	8.01344E-02
Cldn2	Claudin 2	High LOB versus MOB expression		-7.18 (LOB vs MOB)	3.68773E-02
Agt	Angiotensinogen (serpin peptidase inhibitor, clade A, member 8)	Low LCT versus LOB expression		-2.54 (LCT vs LOB)	3.60756E-06
Gsn	Gelsolin	Low LCT versus LOB expression		-2.52 (LCT vs LOB)	5.12019E-24
Ppl	Periplakin	Low LCT versus LOB expression		-2.15 (LCT vs LOB)	9.63604E-03
Ttr	Transthyretin	High MOB versus LOB expression		-6.62 (LOB vs MOB)	NA
Cdh2	Cadherin 2	Major regulator inflammation-induced of endothelial to mesenchymal transition and subtle changes in adult Poly I:C vs weanling I:C (as well as vehicle) in Poly I:C RNA-seq	Group 3: Known regulators of BBB (TJ, AJ, GJs)		
Cdh5	Cadherin 5	Important component of blood-brain barrier that is dysregulated			
Cldn1	Claudin 1	Major regulator of blood-brain barrier			
Cldn3	Claudin 3	Expressed in brain microvessels and correlated to alteration of blood-brain barrier			
Cldn5	Claudin 5	Major regulator of blood-brain barrier			
Gja1	Gap junction protein, alpha 1	connexin , alteration of gap junction pathway in RNA-seq			
Tjp1 (ZO-1)	Tight junction protein 1	Major component of blood-brain barrier			
Tjp2 (ZO-2)	Tight junction protein 2	Major component of blood-brain barrier			
Vegfa	Vascular endothelial growth facto	Regulator of blood-brain barrier in inflammatory conditions and subtle change in adult Poly I:C vs weanling Poly I:C			
Cxcl1	Chemokine (C-X-C motif) ligand	Major regulator of blood-brain barrier and granulocyte adhesion that is induced during neuroinflammation and subtle changes observed comparing adult Poly I:C vs weanling Poly I:C (Poly I:C RNA-seq)	Group 4: Immune regulators		
Ifnb	Interferon beta 1, fibroblast	Immune component that helps stabilizing blood-brain barrier and altered in adult Poly I:C vs adult vehicle (Poly I:C RNA-seq)			
Il-23a	Interleukin 23, alpha subunit p19	Aggravates autoimmune response in brain and high log fold change adult Poly I:C vs weanling Poly I:C (reduced in adults in Poly I:C RNA-seq, low base-mean)			

Supplementary Table 1 (related to Figure 1 and 2). List of genes chosen for siRNA screen. From the RNA-seq analyses described in Figs. 1A-B and S1, and based on known relevance to classical BCEC function, the listed genes were chosen for targeted siRNA screening or other *ex vivo* analyses. Genes are grouped according to their selection criteria which are also indicated. For all the differentially expressed genes identified from RNA-seq analyses, the log 2-fold change and adjusted P values (directly obtained from RNA-seq analyses) are provided in separate columns.

Supplementary Table 2

Gene abbreviation	Gene name	Cell type marker	Average base-mean in *ex vivo* microvessel isolates
Pecam1/ CD31	Platelet/endothelial cell adhesion molecule 1	BCECs	16101
Vcam1	Vascular cell adhesion molecule 1	BCECs	20747
Vwf	Von Willebrand factor	BCECs	20977
Gfap	Glial fibrillary acidic protein	Astrocytes	102
Tubb3	Tubulin, beta 3 class III	Neurons	45
Cspg4	Chondroitin sulfate proteoglycan 4	OPCs, pericytes	1419
Pdgfb	Platelet derived growth factor, B polypeptide	Pericytes	4772
Cnn1	Calponin 1	Smooth muscle cells	614

Supplementary Table 2. RNA seq expression basemean values for cell type specific genes from brain microvessel preparations. The BCEC-specific or other brain parenchymal/ endothelial-specific cell markers base-mean values (representing relative RNA expression level) was obtained from the RNA-seq analyses. These data show that *ex vivo* isolated brain microvessel fragments were highly enriched with BCECs.

Supplementary Table 3				
**Animal number**	**Genotype**	**Infection age**	**Postinfection days**	**Treatment**
Efna2-/- (m)_C_1	Efna2-/-3+/-5+/-	Adult	8	100000 PFU LACV
Efna2-/- (m)_C_2	Efna2-/-3+/-5+/-	Adult	8	100000 PFU LACV
Efna2-/- (m)_C_3	Efna2-/-3-/-5+/-	Adult	8	100000 PFU LACV
Efna2-/- (m)_C_4	Efna2-/-3+/-5+/-	Adult	8	100000 PFU LACV
Efna2-/- (m)_C_5	Efna2-/-3+/-5+/+	Adult	9	100000 PFU LACV
Efna2-/- (m)_C_6	Efna2-/-3+/-5+/-	Adult	10	100000 PFU LACV
Efna2-/- (m)_C_7	Efna2-/-3+/-5-/-	Adult	11	100000 PFU LACV
Efna2-/- (m)_C_8	Efna2-/-3-/-5+/+	Adult	15	100000 PFU LACV
Efna2-/- (m)_NC_1	Efna2-/-3+/+5+/+	Adult	22	100000 PFU LACV
Efna2-/- (m)_NC_2	Efna2-/-3+/-5+/-	Adult	22	100000 PFU LACV
Efna2-/- (m)_NC_3	Efna2-/-3-/-5+/-	Adult	22	100000 PFU LACV
Efna2-/- (m)_NC_4	Efna2-/-3-/-5+/-	Adult	22	100000 PFU LACV
Efna2-/- (m)_NC_5	Efna2-/-3-/-5+/-	Adult	22	100000 PFU LACV
Efna2-/- (m)_NC_6	Efna2-/-3+/-5+/-	Adult	22	100000 PFU LACV
Efna2-/- (m)_NC_7	Efna2-/-3+/-5+/-	Adult	22	100000 PFU LACV
Efna2-/- (m)_NC_8	Efna2-/-3-/-5+/-	Adult	22	100000 PFU LACV
Efna2-/- (m)_NC_9	Efna2-/-3-/-5+/-	Adult	22	100000 PFU LACV
Efna2-/- (m)_NC_10	Efna2-/-3+/-5+/-	Adult	22	100000 PFU LACV
Efna2+/+ (WT)_C_1	Efan2+/+3+/+5+/+	Adult	8	100000 PFU LACV
Efna2+/+ (WT)_C_2	Efan2+/+3+/+5+/+	Adult	10	100000 PFU LACV
Efna2+/- (m)_NC_1	Efna2+/-3+/-5-/-	Adult	22	100000 PFU LACV
Efna2+/- (m)_NC_2	Efna2+/-3+/-5+/-	Adult	22	100000 PFU LACV
Efna2+/- (m)_NC_3	Efna2+/-3+/+5+/-	Adult	22	100000 PFU LACV
Efna2+/- (m)_NC_4	Efna2+/-3+/+5+/-	Adult	22	100000 PFU LACV
Efna2+/- (m)_NC_5	Efna2+/-3+/+5+/+	Adult	22	100000 PFU LACV
WT_NC_1	Efna2+/+3+/+5+/+	Adult	22	100000 PFU LACV
WT_NC_2	Efan2+/+3+/+5+/+	Adult	22	100000 PFU LACV
WT_NC_3	Efan2+/+3+/+5+/+	Adult	22	100000 PFU LACV
WT_NC_4	Efan2+/+3+/+5+/+	Adult	22	100000 PFU LACV
WT_NC_5	Efan2+/+3+/+5+/+	Adult	22	100000 PFU LACV
WT_NC_6	Efan2+/+3+/+5+/+	Adult	22	100000 PFU LACV
WT_NC_7	Efna2+/+3+/+5+/+	Adult	22	100000 PFU LACV
WT_NC_8	Efna2+/+3+/+5+/+	Adult	22	100000 PFU LACV
WT_NC_9	Efna2+/+3+/+5+/+	Adult	22	100000 PFU LACV
WT_NC_10	Efna2+/+3+/+5+/+	Adult	22	100000 PFU LACV
WT_NC_11	Efna2+/+3+/+5+/+	Adult	22	100000 PFU LACV
WT_NC_12	Efna2+/+3+/+5+/+	Adult	22	100000 PFU LACV
WT_NC_13	Efna2+/+3+/+5+/+	Adult	22	100000 PFU LACV
WT_NC_14	Efna2+/+3+/+5+/+	Adult	22	100000 PFU LACV

*22=Experimental endpoint
C=Clinical
NC=Nonclinical
WT=Wildtype
Efna2 -/- (m)=Efna2 mixed KO
Efna2+/- (m)=Efna2 mixed het

Supplementary Table 3 (related to Figure 4). Genotypes of Efna2 (m) mice used in LACV-induced neurologic disease studies. The mixed *Efna3* and *Efna5* genotypes of *Efna2-/-* and *Efna2+/-* mice that were tested for LACV-induced (10^5 PFU/mouse) neurologic disease. The animal number notes whether mice showed a clinical (C) or nonclinical (NC) phenotype and a postinfection day of <22 indicates the time of clinical symptom onset.

Supplementary Table 4

Other abbreviations	Full names and small descriptions
4-PBA	4-phenylbutyric acid
ACT	Adult cortex BCECs (LACV infected)
AJ	Adherens junction
AL	Adult LACV-infected BCECs
AM	Adult mock-infected BCECs
AOB	Adult olfactory bulb BCECs (LACV infected)
AON	Anterior olfactory nucleus
API	Adult poly I:C stimulated
AV	Adult vehicle
BBB	Blood-brain barrier
BCECs	Brain capillary endothelial cells
BSA	Bovine serum albumin
C	Clinical mice
CJ	Cell junction
CLS2	Collagenase
CNS	Central nervous system
CMC	Carboxymethyl cellulose
CT	Cortex
Cx43	Connexin43, protein synthesized from Gja1
DMEM	Dulbecco's modified Eagle's medium
dpi	Days post-infection
Efn	Ephrin molecule
Efna2-/- (m)	Mixed deficiency genotypes for *Efna3* and *Efna5* with *Efna2-/-*
Efna2-/- (s)	*Efna2-/-3+/+5+/+* mice (Efna2 single knockout)
EphA	EphrinA class receptors
ERp29	Endoplasmic reticulum (ER) protein of 29 kDa
GJ	Gap junction
hpi	Hours potinfection
IC	Intracerebral
IHC	Immunohistochemistry
IP	Intraperitoneal
IPA	Ingenuity Pathway Analysis
IV	Intravenous (retroorbital) injection
LACV	La Crosse virus
LCT	LACV-infected cortical BCECs (weanling)
LOB	LACV-infected olfactory bulb BCECs (weanling)
MCT	Mock-infected cortical BCECs (weanling)
MOB	Mock-infected olfactory bulb BCECs (weanling)
MOI	Multiplicity of infection
NBF	Neutral buffered formalin
NC	Non-clinical
OB	Olfactory bulb
PFA	Paraformaldehyde
Poly I:C	Polyinosinic: polycytidylic acid
Rec-EFNA2	Recombinant mouse EFNA2
RNA-seq	RNA sequencing
RT	Room temperature
si-*CltcRab* or si-Downreg. Control	Combination of siRNAs to Clathrin heavy chain and Rab5a
si-*Ifnar* or si-Upreg. Control	Combination of siRNA to *Ifnar1* and *Ifnar2*
si-NT	Nontargeting control siRNA
siRNA	Small interfering RNA
TJ	Tight junction
WCT	CT BCECs from weanling (LACV infected)
WL	LACV-infected weanling
WOB	Weanling olfactory bulb BCECs (LACV infected)
WM	Weanling mock-inoculated BCECs
WPI	Weanling poly I:C stimulated
WT	*Efna2+/+3+/+5+/+* genotype or wildtype
WV	Weanling vehicle

Supplementary Table 4. List of abbreviations. Due to complexity of the terminologies used across the manuscript, a list of abbreviations used is shown.

Supplementary Table 5

SYBR Primers				
Gene name	Forward Primer Name	Sequence	Reverse primer Name	Sequence
Efna2	EFNA2.1_981F	TCCGAGACAAAATCCTTGCT	EFNA2.1_1089R	GTCTTCTCCTCAGGCATTGG
Efna2	EFNA2.2_677F	TTCCCTGGGCTTTGAGTTCC	EFNA2.2_775R	TAAACCTTGAGTCGCAGGCA
H-Q6	H2Q6_265F	AAGGGCCATGAGGAGAGTTT	H2Q6_371R	ACGTCACAGCCATACATCCA
Bst1	Bst1.1_363F	TGAGCTATGGGGAGAACACC	Bst1.1_466R	TCCAGAGGCATTTTCCTGTC
Bst1	Bst1.2_1624F	GCACTACTGCTCAGCCATCA	Bst1.2_1727R	TAGCCATTTGGTCAGGCTTC
Mmp25	Mmp25.1_2625F	ACATTGAACCCCAGCATCTC	Mmp25.1_2739R	CTTGGCCTAAGTCCCTACCC
Mmp25	Mmp25.2_2066F	GTCACTGCGAGCTCAATCAA	Mmp25.2_2174R	CAAGGAAGGACCAGGTTTCA
Clec4e	Clec4e.1_1832F	CCTGCTCCTATCCACCTCAC	Clec4e.1_1926R	TGCATTCAGGAAGCACTGAG
Clec4e	Clec4e.2_1905F	TGCTCAGTGCTTCCTGAATG	Clec4e.2_2013R	ATTGGCTATTGTTGCCTGCT
Cldn1	CLDN1.1_1579F	TTTTCCCGATGACCTTTCTG	CLDN1.1_1687R	CAACAACAGGGTTAGCAGCA
Cldn1	CLDN1.2_1484F	AATTTGGAGCCCACATTTTG	CLDN1.2_1596R	GAAAGGTCATCGGGAAAACA
Aqp1	Aqp1.1_1469F	CTTACCTCCAGGACCCTTCC	Aqp1.2_1568R	CCCTTCCTTGCCACTTTACA
Aqp1	Aqp1.2_915F	CATGAAGGTGTGGACCAGTG	Aqp1.2_1009R	GCCTCCTCTATTTGGGCTTC
Ttr	Ttr.1_531F	TTTCACAGCCAACGACTCTG	Ttr.1_637R	TCTCTCAATTCTGGGGGTTG
Ttr	Ttr.2_193F	GCTTCCCTTCGACTCTTCCT	Ttr.2_305R	GCATCCAGGACTTTGACCAT
Lacv	LACVs.2-552F	ATTCTACCCGCTGACCATTG	LACVs.2-650R	GTGAGAGTGCCATAGCGTTG
Gapdh	Gapdh2-152f	AACGACCCCTTCATTGAC	Gapdh2-342r	TCCACGACATACTCAGCA

TaqMan Primers	
Gene name	Details
Gja1	Thermo Scientific, Cat no: 4331182, Assay Id: Mm00439105_m1
Gapdh	Thermo Scientific, Cat no: 4331182, Assay Id: Mm99999915_g1

Supplementary Table 5 (continued)						
Gene Symbol	siRNA ID	Sample ID	Gene ID	Lot Number	Sense siRNA Sequence	Antisense siRNA Sequence
Bst1	65854	ASO2EDRQ	12182	AMO21U0B	GGUAGUGUGAAAAUCCUGGtt	CCAGGAUUUUCACACUACCtt
Ifnb1	67501	ASO2EDS6	15977	AMO21U0B	GGAAAAGCAAGAGGAAAGAtt	UCUUUCCUCUUGCUUUUCCtc
Cdh5	161142	ASO2EDSE	12562	AMO21U0B	CGUGAACCGCCAGAAUGCUtt	AGCAUUCUGGCGGUUCACGtt
Mmp25	284179	ASO2EDSU	240047	AMO21U0B	CGGAAUUGACCUAUUUGCAtt	UGCAAAUAGGUCAAUUCCGtt
Gsn	90887	ASO2EDT2	227753	AMO21U0B	GGCUUAAGGACAAGAAGAUtt	AUCUUCUUGUCCUUAAGCCtg
Cxcl1	61979	ASO2EDTA	14825	AMO21U0B	GGGUGUUGUGCGAAAAGAAtt	UUCUUUUCGCACAACACCCtt
Cxcl10	75389	ASO2EDTI	15945	AMO21U0B	GGAACCUGAAAAUGUAUGUtt	ACAUACAUUUUCAGGUUCCtc
Ifnar1	159104	ASO2EDTQ	15975	AMO21U0B	CCUUUUCAGCAGAAUAUCGtt	CGAUAUUCUGCUGAAAAGGtc
Efna2	61287	ASO2EDRR	13637	AMO21U0B	GGACAGAAUGGAAAUUCUUtt	AAGAAUUUCCAUUCUGUCCtt
Cldn1	72259	ASO2EDRZ	12737	AMO21U0B	GGUGCAGAAGAUGUGGAUGtt	CAUCCACAUCUUCUGCACCtc
Ifna4	67404	ASO2EDS7	15967	AMO21U0B	GUCCUGGAAGAAAUGAGAAtt	UUCUCAUUUCUUCCAGGACtg
Cldn3	66057	ASO2EDSF	12739	AMO21U0B	GGCCAAGAUCACCAUCGUGtt	CACGAUGGUGAUCUUGGCCtt
Tjp2	69623	ASO2EDSN	21873	AMO21U0B	GGUUUUGAAGUGAUUGAAGtt	CUUCAAUCACUUCAAAACCtc
Il23a	175086	ASO2EDSV	83430	AMO21U0B	GCGGGACAUAUGAAUCUACtt	GUAGAUUCAUAUGUCCCGCtg
Tjp1	65097	ASO2EDT3	21872	AMO21U0B	GGCAAGUUAAAAAUGGUAGtt	CUACCAUUUUUAACUUGCCtt
Ifitm3	162876	ASO2EDTB	66141	AMO21U0B	CCUUCACACUUAAUAGAGGtt	CCUCUAUUAAGUGUGAAGGtt
Ly6c2	500170	ASO2EDTJ	100041546	AMO21U0B	CAUGUGUGCCUGUCAAUGAtt	UCAUUGACAGGCACACAUGgg
H2-Q6	283299	ASO2EDRS	110557	AMO21U0B	CGUAGCUAUUCUGGUUGUCtt	GACAACCAGAAUAGCUACGtt
Aqp1	159694	ASO2EDS0	11826	AMO21U0B	GCCCAAAUAGAGGAGGCUUtt	AAGCCUCCUCUAUUUGGGCtt
Irf7	72667	ASO2EDS8	54123	AMO21U0B	GGUGUACGAACUUAGCCGGtt	CCGGCUAAGUUCGUACACCtt
Cdhr1	86319	ASO2EDSG	170677	AMO21U0B	GGGAAGAUGAGAUUGAAGCtt	GCUUCAAUCUCAUCUUCCCtt
Cldn2	72350	ASO2EDSO	12738	AMO21U0B	GGGCAAUUGCUAUAUCUUAtt	UAAGAUAUAGCAAUUGCCCtc
L1cam	62776	ASO2EDSW	16728	AMO21U0B	GGAAGAAUUGGGUGUAGUGtt	CACUACACCCAAUUCUUCCtt
Gja1	67260	ASO2EDT4	14609	AMO21U0B	GGUAUGUAACGGUAUUUAAtt	UUAAAUACCGUUACAUACCtc
Cdh1	161134	ASO2EDTC	12550	AMO21U0B	GCUGUCUACCAAAGUGACGtt	CGUCACUUUGGUAGACAGCtc
Vegfa	240535	ASO2EDTK	22339	AMO21U0B	CGAGAUAGAGUACAUCUUCtt	GAAGAUGUACUCUAUCUCGtc
Il10	67599	ASO2EDRT	16153	AMO21U0B	GGUGAAGAGUGAUUUUAAUtt	AUUAAAAUCACUCUUCACCtg
Ttr	71498	ASO2EDS1	22139	AMO21U0B	GGUAUGCGAUCAAACUUUUtt	AAAAGUUUGAUCGCAUACCtt
Cldn5	100363	ASO2EDS9	12741	AMO21U0B	GGUGUAUGAAUCUGUGCUGtt	CAGCACAGAUUCAUACACCtt
Mmp8	62965	ASO2EDSH	17394	AMO21U0B	GGGCUGAAGUGAAAACAGCtt	GCUGUUUUCACUUCAGCCCtt
Bst1	240537	ASO2EDSP	12182	AMO21U0B	GGCAUUCAUCUAGUCCUGCtt	GCAGGACUAGAUGAAUGCCtt
Ifnb1	67588	ASO2EDT5	15977	AMO21U0B	GGUACCUUAAACUCAUGAAtt	UUCAUGAGUUUAAGGUACCtt
Cdh5	161143	ASO2EDTD	12562	AMO21U0B	GCUAUAGGGACCUCUGUCAtt	UGACAGAGGUCCCUAUAGCtg
Agt	162196	ASO2EDTL	11606	AMO21U0B	CGCUCUCUGGAUUUAUCCAtt	UGGAUAAAUCCAGAGAGCGtg
Ppl	63773	ASO2EDRU	19041	AMO21U0B	GGAACUCUCAGAUCUGAUUtt	AAUCAGAUCUGAGAGUUCCtt
Cdh2	60629	ASO2EDS2	12558	AMO21U0B	GGAUGUGCACGAAGGACAGtt	CUGUCCUUCGUGCACAUCCtt
Ccl11	151582	ASO2EDSA	20292	AMO21U0B	CCAACAACAGAUGCACCCUtt	AGGGUGCAUCUGUUGUUGGtg
Mmp15	63149	ASO2EDSI	17388	AMO21U0B	GGAUGGACAUUUUGUCUUCtt	GAAGACAAAAUGUCCAUCCtg
Efna2	157275	ASO2EDSQ	13637	AMO21U0B	CGACUACCUGGAUAUCUACtt	GUAGAUAUCCAGGUAGUCGtt
Cldn1	72349	ASO2EDSY	12737	AMO21U0B	GGCUAACAUGGUUUUAUAUtt	AUAUAAAACCAUGUUAGCCtg
Ifna4	67497	ASO2EDT6	15967	AMO21U0B	GCCAUCCUUGUGCUAAGAGtt	CUCUUAGCACAAGGAUGGCtt
Cldn3	66149	ASO2EDTE	12739	AMO21U0B	GGGAUUUCUAUAACCCGUUtt	AACGGGUUAUAGAAAUCCCtg
Tjp2	69717	ASO2EDTM	21873	AMO21U0B	GGAUAUCUCGGAAAUAGAGtt	CUCUAUUUCCGAGAUAUCCtc
Mmp25	284178	ASO2EDRV	240047	AMO21U0B	GGAGCCUGACAUCAUUAUCtt	GAUAAUGAUGUCAGGCUCCtg
Gsn	90697	ASO2EDS3	227753	AMO21U0B	GGAGGUGUGGCAUCUGGAUtt	AUCCAGAUGCCACACCUCCtt
Cxcl1	61884	ASO2EDSB	14825	AMO21U0B	GGGUCCUAUUUAUUUAUGUtt	ACAUAAAUAAAUAGGACCCtc
Cxcl10	75217	ASO2EDSJ	15945	AMO21U0B	GGUCUAAAAGGGCUCCUUAtt	UAAGGAGCCCUUUUAGACCtt
H2-Q6	288488	ASO2EDSR	110557	AMO21U0B	GCUUGGCCAUCAUUGGAGCtt	GCUCCAAUGAUGGCCAAGCta
Aqp1	159695	ASO2EDSZ	11826	AMO21U0B	GCUGACCAAAGUCACUCCCtt	GGGAGUGACUUUGGUCAGCtt
Irf7	72762	ASO2EDT7	54123	AMO21U0B	GGAUCAUUUCUGGCAAGAGtt	CUCUUGCCAGAAAUGAUCCtg
Cdhr1	172576	ASO2EDTF	170677	AMO21U0B	GCACUAGAAGUGUCUUUUCtt	GAAAAGACACUUCUAGUGCtg
Cldn2	161757	ASO2EDTN	12738	AMO21U0B	CGAGUUCCUAUGUUGGUGCtt	GCACCAACAUAGGAACUCGtt
Tjp1	64907	ASO2EDS4	21872	AMO21U0B	GGACAGCUACAGGAAAAUGtt	CAUUUUCCUGUAGCUGUCCtt
Ifitm3	78056	ASO2EDSC	66141	AMO21U0B	GGUGUAACACUCAUUUAUCtt	GAUAAAUGAGUGUUACACCtg
Ly6c2	500169	ASO2EDSK	100041546	AMO21U0B	UGCCAAUCAAGGAUCCUAAtt	UUAGGAUCCUUGAUUGGCAca
Il10	159141	ASO2EDSS	16153	AMO21U0B	GCCGGGAAGACAAUAACUGtt	CAGUUAUUGUCUUCCCGGCtg
Ttr	188371	ASO2EDT0	22139	AMO21U0B	CCAAAUCGUACUGGAAGACtt	GUCUUCCAGUACGAUUUGGtg
Cldn5	101985	ASO2EDT8	12741	AMO21U0B	GUGCAAGGUGUAUGAAUCUtt	AGAUUCAUACACCUUGCACtg
Mmp8	63151	ASO2EDTG	17394	AMO21U0B	GGAGAUGCUCAUUUUGAUUtt	AAUCAAAAUGAGCAUCUCCtc
Ifnar1	67499	ASO2EDTO	15975	AMO21U0B	GGCGAAGUGGUUAAAAGUGtt	CACUUUUAACCACUUCGCCtc
L1cam	62683	ASO2EDRX	16728	AMO21U0B	GGACACCAUGUGCUAGAGCtt	GCUCUAGCACAUGGUGUCCtt
Gja1	67075	ASO2EDS5	14609	AMO21U0B	GGAAGAGAAGCUGAACAAGtt	CUUGUUCAGCUUCUCUUCCtt
Cdh1	161133	ASO2EDSD	12550	AMO21U0B	GCGGCAUCUAAAGCUCCACtt	GUGGAGCUUUAGAUGCCGCtt
Vegfa	65293	ASO2EDSL	22339	AMO21U0B	GGCUGCUGUAACGAUGAAGtt	CUUCAUCGUUACAGCAGCCtg
Ppl	63868	ASO2EDST	19041	AMO21U0B	GGUGGAAAGGAACAUUGUGtt	CACAAUGUUCCUUUCCACCtg
Cdh2	160128	ASO2EDT1	12558	AMO21U0B	GCAGGCAAAGUUCCUGAUAtt	UAUCAGGAACUUUGCCUGCtc
Ccl11	151583	ASO2EDT9	20292	AMO21U0B	GGAACACAAUGGGACGAGUtt	ACUCGUCCCAUUGUGUUCCtc
Mmp15	155387	ASO2EDTH	17388	AMO21U0B	GGUCACAGACCAAGCUUAUtt	AUAAGCUUGGUCUGUGACCtg
Ifnar1	67586	ASO2EDTP	15975	AMO21U0B	GGCAUGAACCAUUCAGUAAtt	UUACUGAAUGGUUCAUGCCtg

Supplementary Table 5. List of primers and siRNAs. A list of all the primers and siRNAs used in this study.
