# Supplementary material for: Identification of age-specific gene regulators of La Crosse virus neuroinvasion and pathogenesis
Source: Nat Commun. 2023 May 18;14:2836. doi: 10.1038/s41467-023-37833-x (PMC10195820; doi:10.1038/s41467-023-37833-x)
Supplement: Supplementary file 3 — Reporting Summary [file 41467_2023_37833_MOESM3_ESM.pdf]

nature portfolio

Corresponding author(s): Karin E. Peterson and Iain D.C. Fraser

Last updated by author(s): 2023-19-01

Reporting Summary

Nature Portfolio wishes to improve the reproducibility of the work that we publish. This form provides structure for consistency and transparency in reporting. For further information on Nature Portfolio policies, see our Editorial Policies and the Editorial Policy Checklist.

Statistics

For all statistical analyses, confirm that the following items are present in the figure legend, table legend, main text, or Methods section.

n/a	Confirmed	
☐	☒	The exact sample size (*n*) for each experimental group/condition, given as a discrete number and unit of measurement
☐	☒	A statement on whether measurements were taken from distinct samples or whether the same sample was measured repeatedly
☐	☒	The statistical test(s) used AND whether they are one- or two-sided<br>*Only common tests should be described solely by name; describe more complex techniques in the Methods section.*
☐	☒	A description of all covariates tested
☐	☒	A description of any assumptions or corrections, such as tests of normality and adjustment for multiple comparisons
☐	☒	A full description of the statistical parameters including central tendency (e.g. means) or other basic estimates (e.g. regression coefficient) AND variation (e.g. standard deviation) or associated estimates of uncertainty (e.g. confidence intervals)
☐	☒	For null hypothesis testing, the test statistic (e.g. *F*, *t*, *r*) with confidence intervals, effect sizes, degrees of freedom and *P* value noted<br>*Give P values as exact values whenever suitable.*
☒	☐	For Bayesian analysis, information on the choice of priors and Markov chain Monte Carlo settings
☐	☒	For hierarchical and complex designs, identification of the appropriate level for tests and full reporting of outcomes
☒	☐	Estimates of effect sizes (e.g. Cohen's *d*, Pearson's *r*), indicating how they were calculated

Our web collection on statistics for biologists contains articles on many of the points above.

Software and code

Policy information about availability of computer code

Data collection	Data collection was done using LAS X (For Leica confocal and epifluorescence microscope, version 10), QuantStudio Real-Time PCR software (in a QuantStudio 6 Flex machine, Applied Biosystems).
Data analysis	Thermo Scientific HCS Studio/ Cellomics software (Cx7 machine) Graph pad Prism 8 and 9, Microsoft Excel (2010), Ingenuity Pathway Analysis (IPA, version 84978992, Qiagen)R (3.6.0), Fiji (1.52n), ImageJ (1.52a), Imaris, SIGNAL 2.0 and FlowJo (10.8.1)

For manuscripts utilizing custom algorithms or software that are central to the research but not yet described in published literature, software must be made available to editors and reviewers. We strongly encourage code deposition in a community repository (e.g. GitHub). See the Nature Portfolio guidelines for submitting code & software for further information.

Data

Policy information about availability of data

All manuscripts must include a data availability statement. This statement should provide the following information, where applicable:

- Accession codes, unique identifiers, or web links for publicly available datasets
- A description of any restrictions on data availability
- For clinical datasets or third party data, please ensure that the statement adheres to our policy

The RNA-seq data have been deposited to the Gene Expression Omnibus (GEO) and are publicly available (GSE217434). Hyperlink to the dataset: https://www.ncbi.nlm.nih.gov/geo/query/acc.cgi?acc=Gse217434. The SIGNAL software is available to the public using this hyperlink: https://signal.niaid.nih.gov/. The

wildtype mice (RRID: IMSR JAX:000664 and Strain #:000664) are available in The Jackson Laboratory. The Efna2-/- mice are available upon request in the laboratory of Dr. Iain Fraser, NIAID. The other datasets used and/or analyzed during the current study are available from the corresponding author upon request.

Human research participants

Policy information about studies involving human research participants and Sex and Gender in Research.

Reporting on sex and gender	Not applicable.
Population characteristics	Not applicable.
Recruitment	Not applicable.
Ethics oversight	Not applicable.

Note that full information on the approval of the study protocol must also be provided in the manuscript.

Field-specific reporting

Please select the one below that is the best fit for your research. If you are not sure, read the appropriate sections before making your selection.

☒ Life sciences ☐ Behavioural & social sciences ☐ Ecological, evolutionary & environmental sciences

For a reference copy of the document with all sections, see nature.com/documents/nr-reporting-summary-flat.pdf

Life sciences study design

All studies must disclose on these points even when the disclosure is negative.

Sample size	Sample sizes were chosen in-line with current standards for biological replicates of in-vitro, ex vivo or in vivo work. The sample sizes are described in each individual panels and they have at least 3 datapoints to perform appropriate statistical test (We determined this to be sufficient owing to control conditions applied to the experiments, described in the main manuscript). Prior studies done on La Crosse virus (in Dr. Peterson's lab) and published transcriptomic screens from our labs also helped us decide the sample size.
Data exclusions	Generally data were not excluded except significant outliers (Grubb's test was applied in these cases, which is a common statistical test to remove statistically significant outliers). Examples of data loss include technical errors (for example, drying of medium/ human error/ inability to maintain temperature conditions), loss of cells (due to non-maintenance of appropriate culture conditions or senescent primary cells), loss of tissue section (for example, inability to harvest organs due to abrupt mice death without neurological symptom/ 4-PBA injection-caused acute death of mice/ inability to harvest organs), instrument or software-based inability to acquire images (due to manufacturing faults of imaging plates/ high level of autofluorescence or thickness issues in corner wells of imaging plate) or animals that did not fulfill endpoint criteria described in the study. Overall, all technically sound data points were included.
Replication	Our experiments used ≥3 replicates (mentioned in details in main manuscript) typically across screening, validation and animal studies to provide even greater statistical clarity to our work. All attempts of replications were successful, given they had no technical errors as described in the data exclusions section.
Randomization	No statistical method of randomization was used. The mice were randomly distributed by third party technicians (with the knowledge of age-specification). However all cells and tissues that passed quality control were analyzed equally with no sub-sampling and thus, there was no requirement for randomization.
Blinding	Blinding was conducted for animal studies, where the monitoring of neurologic symptoms were done by a third party technician who was blinded to group allocation. However, blinding was not possible for all experiments as in most cases, the investigators who designed the experiment were also responsible for data processing and analysis. Quantifications were always performed using computational approaches applied equally to all experimental conditions. For imaging analysis, thresholds for defining a nucleus (cell count) or viral intensity were chosen using Cellomics software or FlowJo and applied in an unbiased and equal fashion to all samples.

Reporting for specific materials, systems and methods

We require information from authors about some types of materials, experimental systems and methods used in many studies. Here, indicate whether each material, system or method listed is relevant to your study. If you are not sure if a list item applies to your research, read the appropriate section before selecting a response.

Materials & experimental systems

n/a	Involved in the study
☐	☒ Antibodies
☐	☒ Eukaryotic cell lines
☒	☐ Palaeontology and archaeology
☐	☒ Animals and other organisms
☒	☐ Clinical data
☒	☐ Dual use research of concern

Methods

n/a	Involved in the study
☒	☐ ChIP-seq
☒	☐ Flow cytometry
☒	☐ MRI-based neuroimaging

Antibodies

Antibodies used

1. anti-LACV antibody (mosue and rabbit), hyperimmune or polyclonal sera purified in lab
2. ZO1 polyclonal antibody (rabbit, Invitrogen/Thermo Scientific, Cat no: 61-7300, Lot no: SA243690)
3. Cx43 Polyclonal antibody (rabbit, Sigma Aldrich/ Merck Millipore, Cat no: C6219, Lot no: 027M480V)
4. GAPDH polyclonal antibody (rabbit, AbCam, Cat no: ab8245, Lot no: GR3424359-I)
5. Phalloidin Alexa Fluor 594 (Invitrogen, Cat no: A12381, Lot no: 2161933)

All the antibodies are described in material and methods section.

Validation

1. Anti-LACV antibody is validated in several prior studies in Dr. Peterson's lab (Winkler et al, Acta Neropath, 2015, Ojha et al., Nat. Micro., 2021, Basu et al, J. Neuroinflamm., 2021).
2. Manufacturer mentions "61-7300 has been successfully used in Western blot, Immunoprecipitation, Immunofluorescence, ELISA and Immunohistochemistry." (https://www.thermofisher.com/antibody/product/ZO-1-Antibody-Polyclonal/61-7300).
3. Manufacturer mentions "Anti-Connexin-43 may be used in immunoblotting, immunocytochemistry and immunohistochemistry (frozen and formalin-fixed, paraffin-embedded tissues)." (https://www.sigmaaldrich.com/US/en/product/sigma/c6219).
4. Manufacturer mentions "Our Abpromise guarantee covers the use of ab8245 in the following tested applications. WB: 1/500 - 1/10000. Detects a band of approximately 36 kDa (predicted molecular weight: 40.2 kDa." (https://www.abcam.com/products/primary-antibodies/gapdh-antibody-6c5-loading-control-ab8245.html)
5. Manufacturer mentions "Alexa Fluor™ 594 phalloidin can be used to visualize and quantitate F-actin in tissue sections, cell cultures, or cell-free preparations." (https://www.thermofisher.com/order/catalog/product/A12381)

Eukaryotic cell lines

Policy information about cell lines and Sex and Gender in Research

Cell line source(s)

Cell line, bEnd.3 (CRL-2299) was purchased from ATCC and primary cultures were established in the laboratory as described in material and methods section.

Authentication

No additional authentication method was used.

Mycoplasma contamination

No additional mycoplasma testing was performed.

Commonly misidentified lines (See ICLAC register)

None used.

Animals and other research organisms

Policy information about studies involving animals; ARRIVE guidelines recommended for reporting animal research, and Sex and Gender in Research

Laboratory animals

Mus musculus, C57Bl6, male or female, 3 weeks (weanling) >6 weeks (adult). This applies for both of the WT and Efna2-/- (m) and Efna2-/- (s) mice. The temperature range for both inside the animal cage and the animal holding room is 69-75 degrees F and the humidity range is 30-70%. A 12 hours light/ dark cycle (6am-6pm) is maintained throughout the year.

Wild animals

None used.

Reporting on sex

No sex-related phenotype was observed, similar to those of prior LACV studies.

Field-collected samples

None.

Ethics oversight

All animal studies were conducted under animal protocol RML-2018-018-E, LISB 3E and LISB 4E, adhering to the Principles of Laboratory Animal Care and in accordance and approval by the NIH/NIAID/RML Institutional Animal Care and Use Committee. No human samples were used in this study.

Note that full information on the approval of the study protocol must also be provided in the manuscript.
